# Phase-Dependent Thermoluminescence Properties of Zinc Sulphide

**DOI:** 10.1007/s10895-024-04050-8

**Published:** 2024-12-03

**Authors:** Shrouk Farouk, Amany Farouk, Nabil El-Faramawy, Nancy N. Elewa

**Affiliations:** Physics Department, Faculty of Science, Ain Shams University, Cairo, Egypt

**Keywords:** Zinc sulphide, Thermoluminescence, XRD, Thermal treatment, Activation energies

## Abstract

In the current study, the effects of heat treatment on the thermoluminescence (TL) and structural properties of zinc blend samples were studied. The samples were annealed after being heated for different time periods from 2 up to 10 h. The wurtzite hexagonal phase was observed upon heat treatment. It was observed that the presence of hexagonal phase with small percentage enhanced the thermoluminescence effect where the highest intensity was found to be for the sample with 8.2% wurtzite and 91.8% cubic zinc sulphide. The morphology of ZnS particles was studied by the HR-TEM analysis and it displayed an aggregated ZnS nanoparticles with almost uniform shape and size. The TL-glow curves for each sample were analysed using the Computerized Glow Curve Deconvolution (CGCD) and initial rise (IR) methods. Six trapping peaks exist between 0.98 and 1.45 eV through the deconvolution process. The number of peaks increased to 8 peaks for the fourth treatment, located between 0.95 and 1.56 eV. The activation energies and the frequency factors for getting traps were evaluated too.

## Introduction

Recent years have seen a significant amount of interest in semiconductor nanomaterials having luminous properties due to their potential use in optoelectronic devices, light emitters, and solar cells. Among these materials, zinc sulphide is considered a promising material that can be utilized for various applications in optical devices such as light-emitting diodes, lasers, flat-panel displays, infrared windows, sensors, and photocatalysts [1, 2]. Zinc sulphide has two stable phases: a cubic zinc blend (ZB) phase and a hexagonal wurtzite (WZ) phase. Both phases are of industrial importance; however, the more stable polymorph is the cubic phase under ambient conditions because of its free energy difference of ∼10 kJ mol^− 1^. The ZB structure’s bandgap is 3.68–3.72 eV, whereas the WZ phase’s is 3.77 eV [1]. The hexagonal polymorph of the ZnS possesses much attention since its luminescent properties are considerably superior to the cubic phase. Therefore, the hexagonal wurtzite structure is preferable in optoelectronics applications [3].

Under the exposure of ionizing radiation, free electrons or holes are created and may be trapped by some defects or centres inside the material. If these traps are energetically deep enough, the trapped carriers may remain stable for a period, unless they are freed by external stimulation. The thermally stimulated light emission is commonly referred to as the thermoluminescence (TL) property of this material. One of the most widely used techniques for studying optical properties is thermo-stimulated luminescence (TL); it is based on creating traps to catch the charge carriers moving to/from the conduction band. As electrons gain energy from radiation and are excited by the conduction band, charge carriers will get caught by the defect traps in the crystalline structure. TL phenomena are present in several naturally occurring minerals, such as muscovite [4], quartz [5–7], feldspar [8], fluorapatite [9], and calcite [10, 11]. This property can be induced synthetically by adding rare elements and few metals to the host material [12–15]. There are various reports of nano-zinc sulphide phase transformation from its cubic to wurtzite phase by annealing [16, 17]. Any change in the crystal structure is of great importance and would influence the TL glow curves [18, 19].

The current work studies the heat treatment effect on the zinc sulphide phase transformation and crystal structure and its influence on luminescence properties and optical sensitivity.

## Materials and methods

The zinc sulphide phase was prepared by using the simple chemical co-precipitation method at room temperature, both zinc sulphate [ZnSO_4_.xH_2_O] and sodium sulphide [Na_2_S.xH_2_O] were used as starting materials to get raw zinc and sulphur. To achieve complete dissolution, 71.9 gm and 19.5 gm of zinc sulphate, and sodium sulphide, respectively, were dissolved in 250 ml of distilled water at room temperature and stirred for 15 min. The mixture of the homogeneous solutions of zinc sulphate and sodium sulphide was placed on a magnetic stirrer for 3 h with continuous stirring at room temperature. The mixture of solutions was kept at room temperature for 24 h before being filtered. The precipitated material was filtered and washed by distilled water several times and then allowed to dry in an oven (120 °C) for 6 h.

The prepared samples were exposed to beta particles at doses ranging from 0.11 Gy to 330 Gy. The irradiation process was carried out using an automated Lexsyg Smart TL/OSL luminescent reader with a built-in source of the ^90^Sr/^90^Y at a dose rate of 0.11 Gy/s and a maximum energy of 2.2 MeV. The glow curves of the prepared samples were deconvoluted using new TL software [20] developed in the Lab of Nuclear Radiation Measurements at the Department of Physics, Faculty of Science, Ain Shams University, Cairo, Egypt.

The texture and morphological properties of the samples were studied using high-resolution transmission electron microscopy (HR-TEM) Talos F200i (FEI, USA) microscope operating at 200 kV. The crystal structure of the samples was studied by x-ray diffraction method (XRD) using an X-ray diffractometer by PANalytical (Model-X’Pert Pro) with CuK_α_ irradiation of wavelength 1.54060 Å with operation voltage of 40 kV and current of 40 mA in a scanning range (2θ) of 20^ο^ − 70^ο^. All the data as well as the blank sample were studied and fitted using Rietveld refinement using the FULLPROF suite [21, 22].

## Results and Discussion

### Morphological and Structural Analysis

**Fig. 1:**
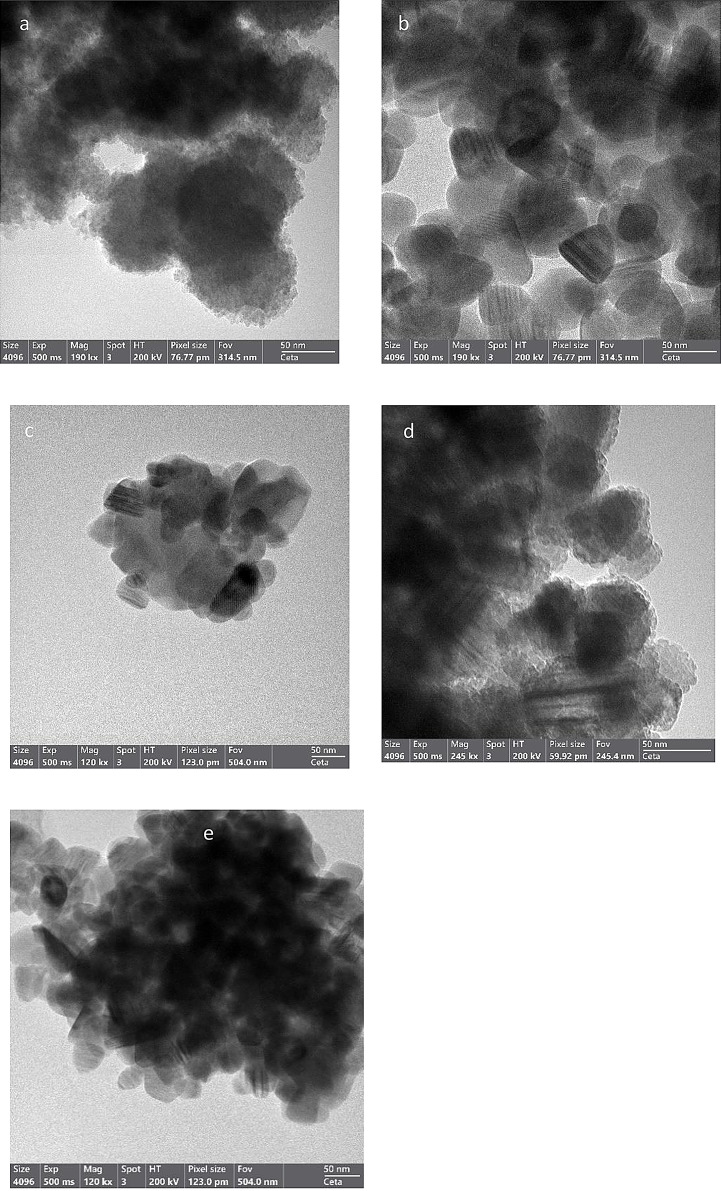
TEM images of the unannealed ZnS (**a**), heat treated ZnS for 2 h (**b**), 4 h (**c**), 6 h (**d**), and 8 h (**e**)

The morphology and structure of ZnS particles were studied by the HR-TEM analysis and XRD. The TEM images of the heat-treated samples in Fig. 1 (b-e) displayed an aggregated ZnS nanoparticles with almost uniform shape and size. It can be observed that the particles are close to the spherical shape, which in turn confirms the new hexagonal phase formation as determined from x-ray diffraction data. At a higher magnification, the lattice spacing of the hexagonal ZnS can be observed for the heat-treated samples, whereas the unannealed sample image (Fig. 1a) shows an aggregated plate-like morphology [23].

**Fig. 2:**
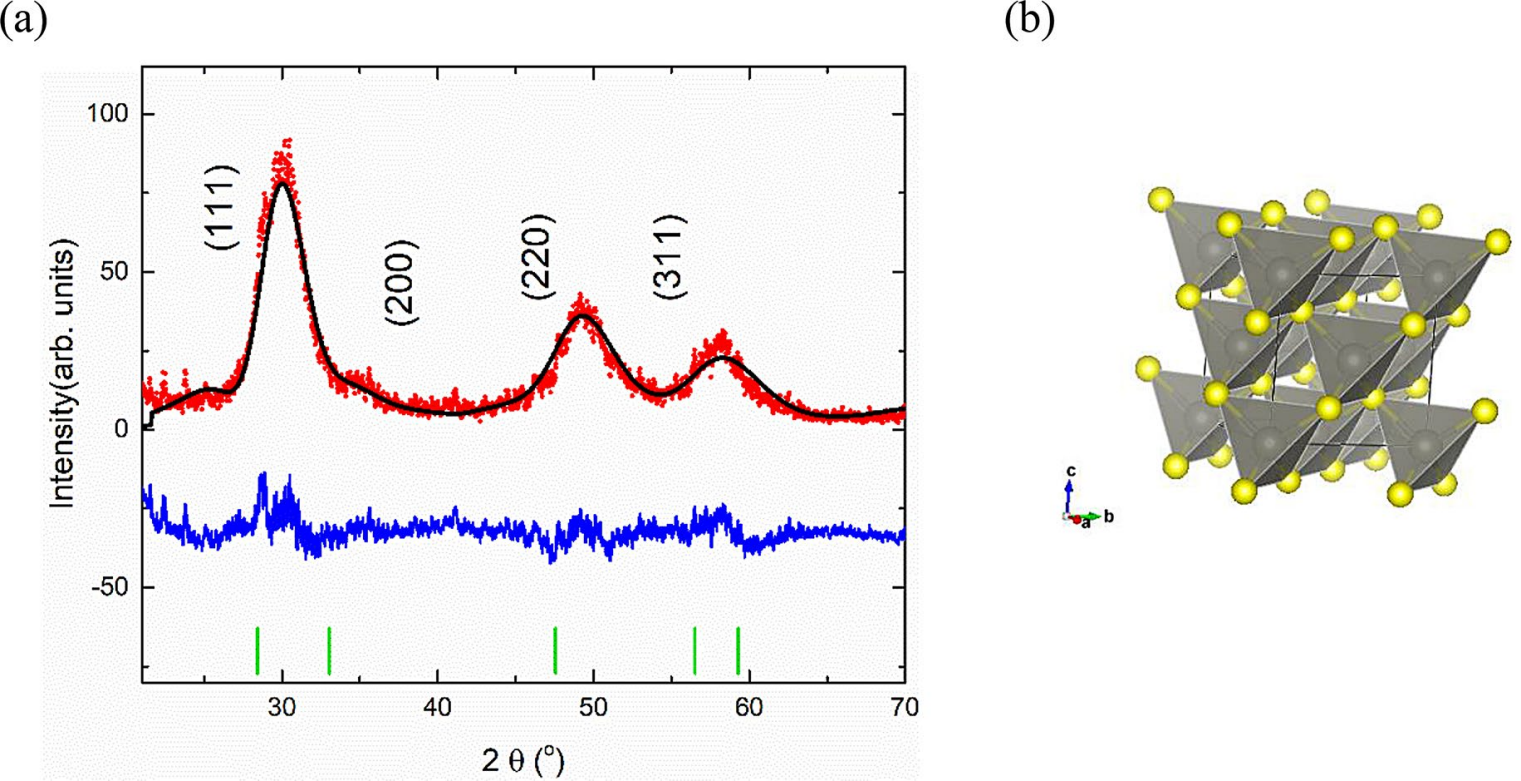
(**a**) Refined X-ray diffraction pattern of the unannealed ZnS: observed (red points), and calculated (-), the difference curve (blue line) and Bragg reflections. (**b**) The cubic crystal structure of the unannealed ZnS with zinc tetrahedra

There are two widely accessible phases of zinc sulphide (ZnS): a zinc blend (ZB) phase and a wurtzite (WZ) phase. In both structures, Zn and S atoms have 4:4 arrangements and are tetrahedrally bonded, but they differ only in the stacking sequence [24]. In zinc blende, the S forms cubic close packing with Zn in the ABCABCA… arrangement, while in the wurtzite phase, the sulphide ions have a hexagonal close packing of ABABAB… [22]. The cubic phase is the stable one at low temperatures and crystallizes in the face-centered cubic space group F-4 3 m. The Rietveld refinement of the XRD data for the pure phase confirms this cubic structure for the unannealed zinc sulphide, as shown in Fig. 2 (a, b).

The results of the X-ray diffraction data show that the unannealed ZnS sample (blank) crystallized in a cubic phase structure with a lattice parameter of 5.3486 (13) (Å). The XRD peaks of the cubic phase are broadened due to the nanocrystalline nature of the synthesized sample. The sample average grain size was calculated using Debye-Scherrer Eq. (1), taking into consideration the instrumental contribution [25], as shown in Table 1.

1 $$\:L=\:\frac{k\:\lambda\:}{\beta\:\:\text{\:cos}\left(\theta\:\right)}$$

Where L is the crystallite size, λ is the wavelength used, β is the integral breadth after correcting the instrument peak broadening (β expressed in radians), and the constant k is a function of the crystallite shape.

**Fig. 3:**
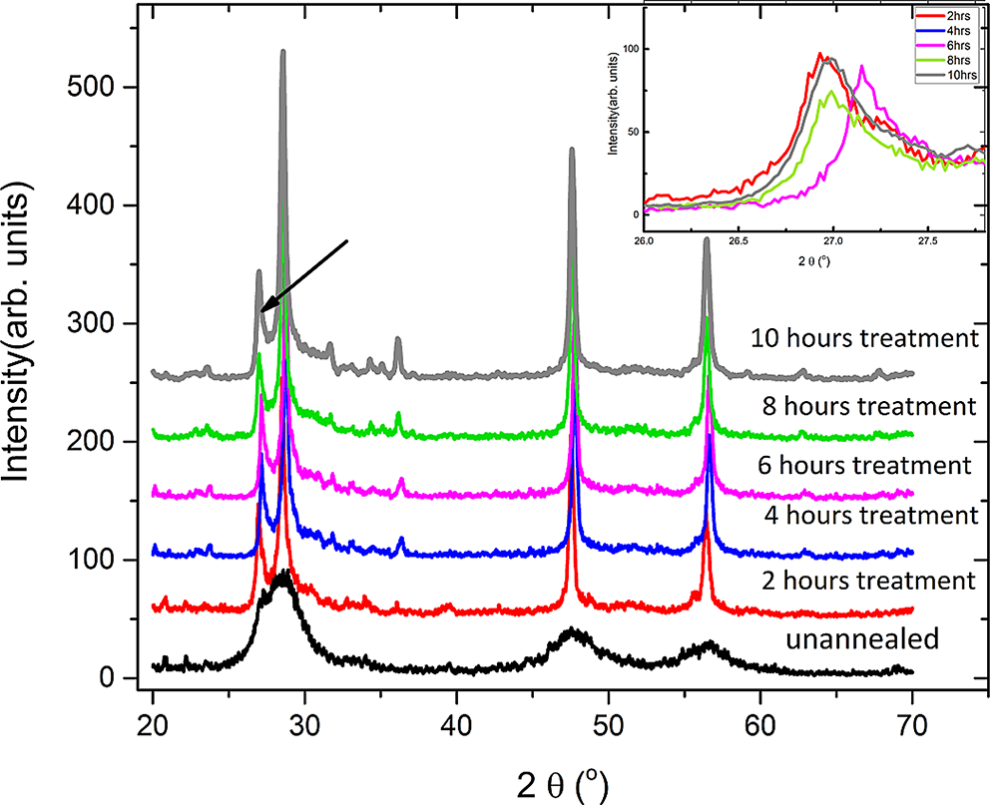
The XRD data from the unannealed sample as a reference and different heat-treated samples, in the annealed samples the hexagonal phase peak starts to appear indicated by the black arrow. The inset figure shows the (100) peak of the hexagonal phase

**Fig. 4:**
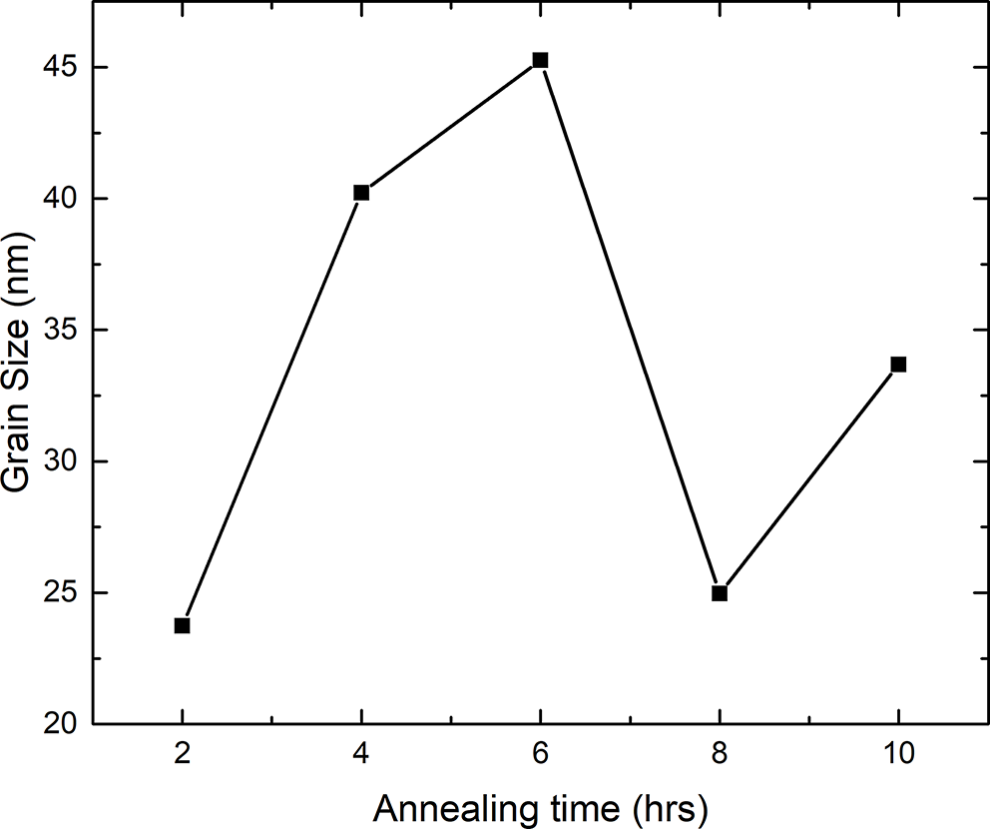
The grain size of the samples with respect to the treatment annealing time

It can be observed from the x-ray diffraction data that there are observable additional peaks as well as a change in those peaks’ intensities upon heat treatment. There is an observed peak found at 26.9^ο^ that corresponds to the (100) peak of the hexagonal phase (Fig. 3). The XRD data shows that the hexagonal phase percentage tends to decrease as the heat treatment period increases from two to eight hours, as seen in Fig. 3 inset. The area percentage for the hexagonal peaks decreases from 12.6% for the 2 h annealed sample to 8.2% for the 8 h annealed samples and increased back for 11.8% for 10 h annealed sample. However, the hexagonal phase shows no significant decrease for samples treated for four and six hours, and the hexagonal peak area percentages were 11.6% and 11%, respectively. This trend is also observed for the grain size as the sample with lowest grain size is the one annealed for 8 h as shown in Fig. 4.

### Thermal Treatment Effect

**Fig. 5:**
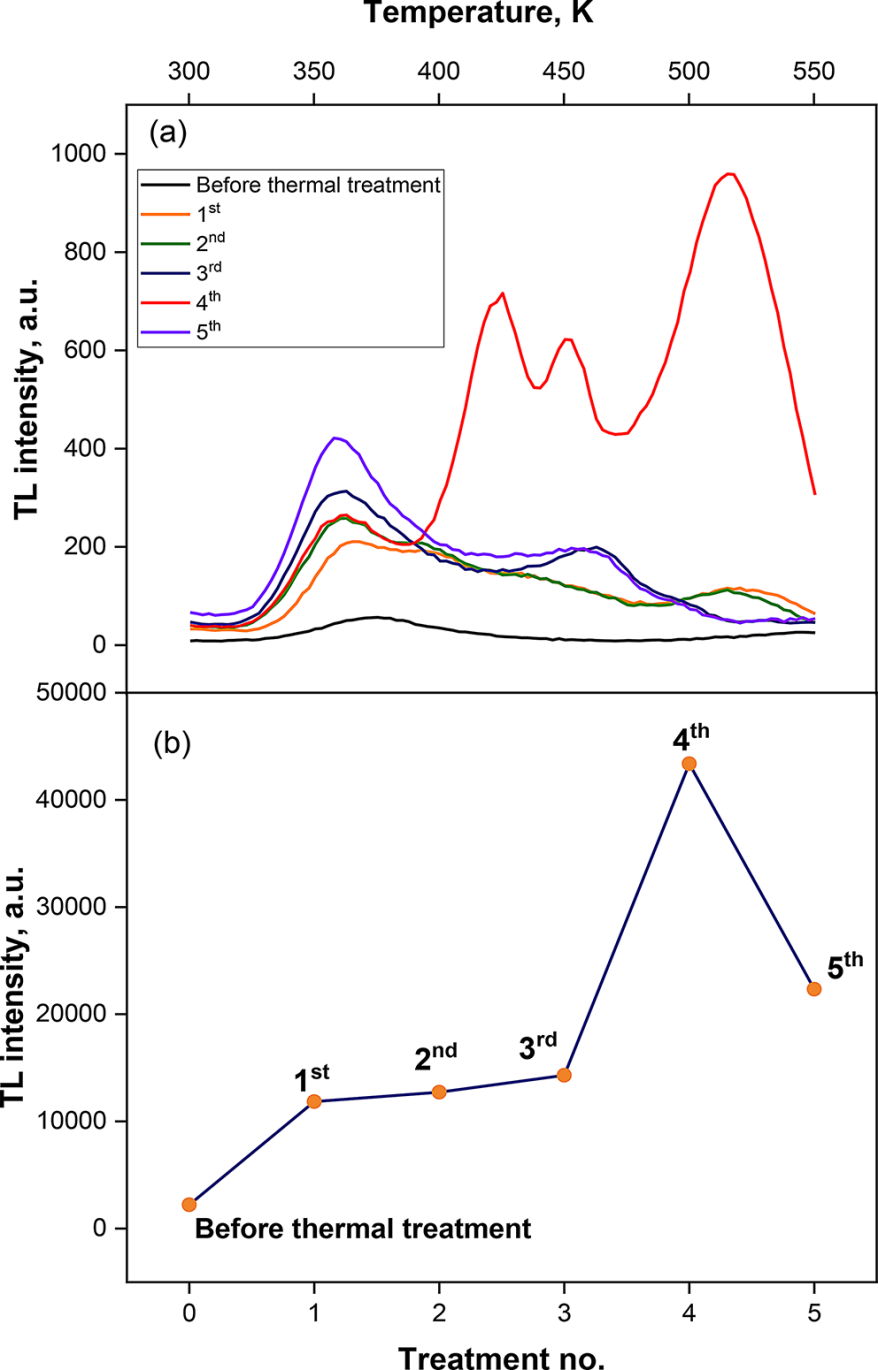
(**a**)The glow curves for the prepared zinc sulphide without thermal treatment and for all the fifth thermal treatment, and (**b**) total area under the obtained glow curves for the prepared zinc sulphide without thermal treatment and for all the fifth thermal treatment

Zinc sulphide (ZnS) powder samples were synthesized and subjected to thermal treatments to increase their TL sensitivity. The powder samples had four different thermal treatments for two, four, six, and eight hours at 500^o^C. Zinc sulphide powder TL glow curves for all four thermal treatments and for the powder without any heat treatment (blank sample) are shown in Fig. 5(a). Regarding the five different heat treatments, it was noticed that the low TL intensity of the light curve was untreated, and up until the fifth one, TL behaved like it was growing up, then decreased again in the fifth treatment. The behaviour is correlated to the appearance of the new hexagonal as it was found that with increasing the annealing time the new hexagonal phase percentage decreases then increased again for the fifth treatment as shown in Table 1. This indicates that the mixture of both phases increased the luminescence properties rather than the inner dislocation in the samples, and the best TL intensity was for the 8.2% hexagonal and 91.8% cubic phase, as presented in Fig. 5(b). Each reading represented the total area beneath the produced glow curves for every thermal treatment. Samples of zinc sulphide (ZnS) powder without heat treatment had the lowest behaviour progressively improving towards the third treatment. According to the figure, the fourth thermal treatment had the highest intensity. For in-depth examinations of the TL characteristics and evaluations of the kinetic parameters, samples of powder zinc sulphide that underwent all thermal treatments will be chosen.

**Table 1:** Area of the hexagonal peak and grain size for the samples at different annealing time

Annealing time hrs	Area of hexagonal peak (%)	Grain size (nm)	Dislocation density (x10^− 4^)
2	12.64	23.74	17.7
4	11.64	40.23	6.2
6	11.60	45.26	4.9
8	8.223	24.97	16
10	11.77	33.68	8.8

### (Tmax – Tstop) and INITIAL RISE (IR) Methods

The ***(T***_***max***_***- T***_***stop***_***)*method** explains how to gradually *empty* the carrier traps and remove their signal from the indicated glow curve. This procedure occurred in many steps; the first step of the procedure: the TL signal of an irradiation sample is recorded (first TL reading) at the temperature raising from room temperature to a suitable low temperature T_stop_. The second and third processes, which involve cooling the irradiation sample that was recorded then the remaining part of the cooled sample glow curve is read (second TL reading). The temperature corresponding to the first maximum intensity in the TL glow curve of the cooled samples (second TL reading) was measured and known as T_max_. Repeating the previous steps with increasing T_stop_ with a temperature step of 5 K each cycle. Finally, the total number of TL components can be evaluated by plotting the values of T_stop_ vs. T_max_.

**Fig. 6:**
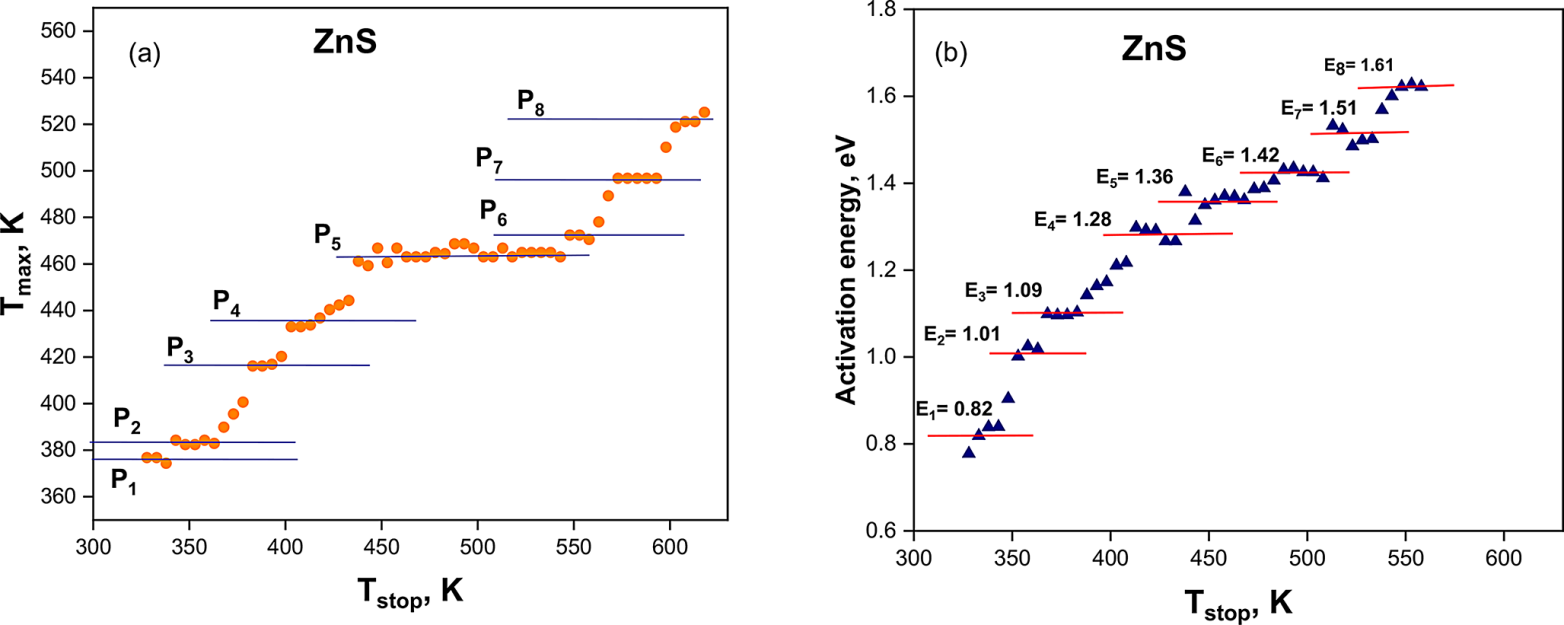
(**a**) (T_max_ – T_stop_) plateau after irradiating the prepared zinc sulphide samples to a beta radiation dose of 5.5 Gy, and (**b**) activation energy against T_stop_ (K) of the prepared zinc sulphide samples using the IR technique

The ***initial rise (IR)*method** of the glow curve analysis and calculating the activation energies experimentally was suggested first by Garlick and Gibson [26]. The amount of the trapped carriers can be assumed to be constant in this early rise region of the glow curve, since the dependence of *n(T)* on temperature is negligible in this region. By using the assumption of *n(T)* is constant and independent of temperature, the intensity of TL is proportional to exp (E/kT) [27]. For this measurement, the synthesized Zinc sulphide was exposed to a beta source radiation of 5.5 Gy then raising T_stop_ from room temperature to 623 K with 5 K increment and for each cycle T_max_ was recorded. Figure 6(a) shows the plateau of T_max_ vs. T_stop_ of the prepared Zinc sulphide samples which predicted the glow curve composed of eight overlapping peaks. Plotting the logarithm of the first rising part of the second TL curve against T_stop_ to provide the IR method and determine the activation energies of the carrier traps. Figure 6(b) illustrates there were eight thermal activation energies (E) ranging from 0.82 to 1.61 eV.

### Glow Curve Analysis and Kinetic Parameters

The activation energies of each of the distinct peaks and their corresponding frequency factors were derived from the deconvolution analysis executed on the TL-glow curves using the newly TL software using the following glow curve deconvolution equation [20]:

2 $$\begin{gathered}\:I\left( T \right) = {I_M}{\text{exp}}\left( {\frac{E}{{k\:T}}\left( {\frac{{T - {T_M}}}{{{T_M}}}} \right)} \right) \hfill \\\times \:{\left[ {1 + \frac{{E\left( {b - 1} \right)\left( {F\left( {T,E} \right) - F\left( {{T_M},E} \right)} \right)}}{{k\:T_M^2\:b{\text{exp}}\left( {\frac{{ - E}}{{k\:{T_M}}}} \right)}}} \right]^{\frac{{ - b}}{{b - 1}}}} \hfill \\ \end{gathered}$$

Here, the parameters I_M_ and T_M_ refer to the maximum intensity of the glow peak and the corresponding temperature, respectively. The optimization parameter E (eV) represents the thermal activation energy of the corresponding carrier trap, b is the order of kinetics, T (K) is the absolute temperature, and k (eV/K) is the Boltzmann constant (8.617E-5 eV/K).

The two functions $\:\text{F}\left(\text{T},\text{E}\right)$ and $\:\text{F}\left({\text{T}}_{\text{M}},\text{E}\right)$ are given by,

3 $$\:F\left(T,E\right)=T\text{exp}\left(\frac{-E}{k\:T}\right)+\:\frac{E}{k}\left(Ei\left(\frac{-E}{k\:T}\right)\right)$$

4 $$\:F\left({T}_{M},E\right)=T\text{exp}\left(\frac{-E}{k\:{T}_{M}}\right)+\:\frac{E}{k}\left(Ei\left(\frac{-E}{k\:{T}_{M}}\right)\right)$$

Such that, Ei(-x), with x > 0, is the exponential integral function [28].

The frequency factor (s) be computed after that using the equation below:

5 $$\:s=\:\left(\frac{E\:\beta\:}{{k\:T}_{M}^{2}}\right)\frac{1}{1+(b-1)\left(\frac{2\:k\:{T}_{M}}{E}\right)}\text{exp}\left(\frac{E}{k\:{T}_{M}}\right)$$

The figure of merit (FOM) that Eddy and Balian created [29]:

6 $$\:FOM=\sum\:_{{j}_{i}}^{{j}_{f}}\frac{100\:({y}_{j}-y({k}_{f}\left)\right)}{A}$$

**Fig. 7:**
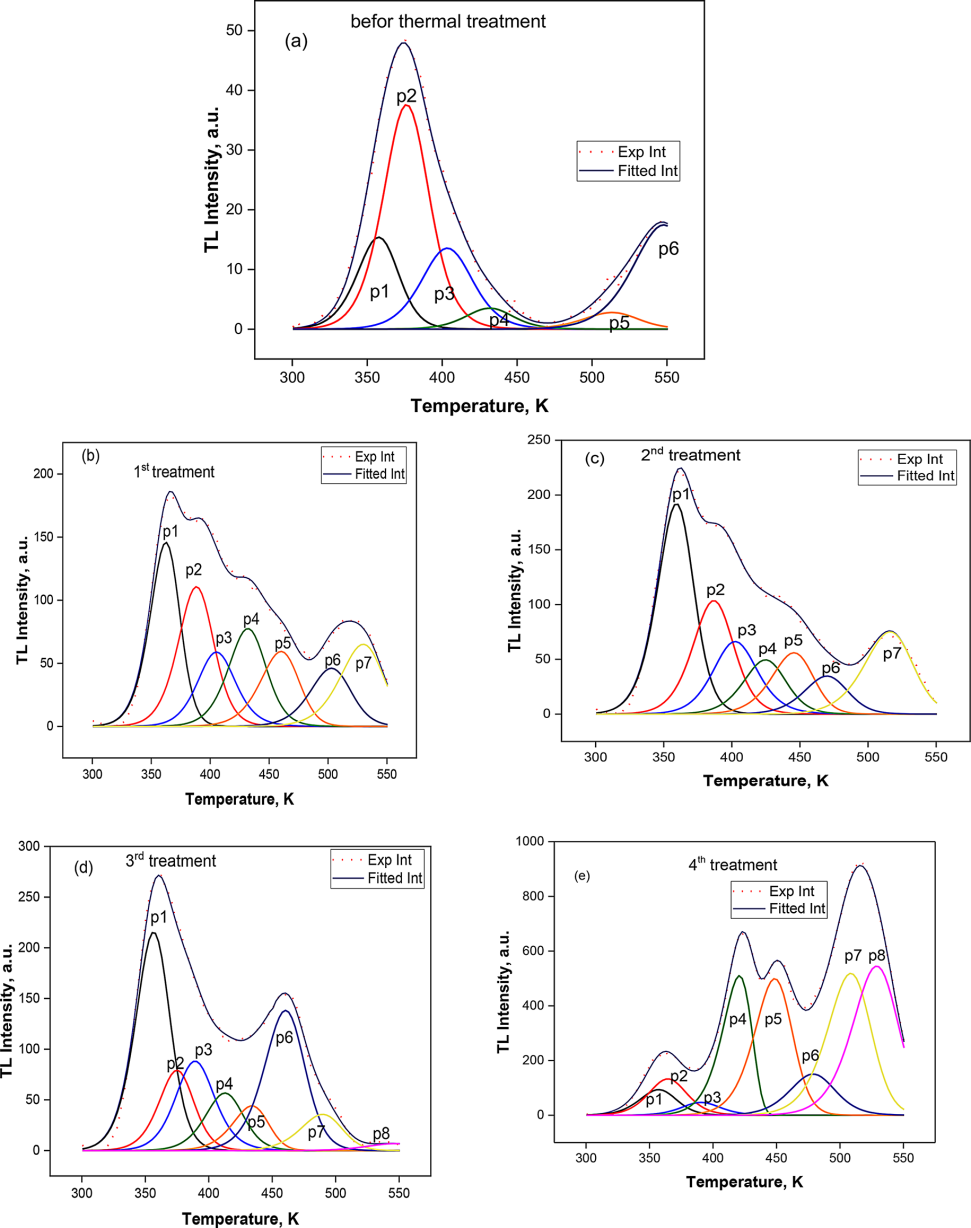
(**a**) The CGCD deconvolution of the glow curve before thermal treatment (blank) of prepared zinc sulphide samples. (**b**, **c**, **d**, **e**) The CGCD deconvolution of the glow curve for 1st, 2nd, 3rd and 4th thermal treatment of prepared zinc sulphide samples

The following channels represent the region of interest: *j*_*i*_ for the first channel, *j*_*f*_ for the final, *y*_*j*_ for the information for the content of channel *j*, *y*(*k*_*f*_) for the fitting function in channel *j*, and *A* for the integral in the interest region of the fitted glow curve.

The zinc sulphide sample produced without thermal treatment (blank) and the sample that received a fourth thermal treatment after being subjected to 1.0 KGy had their TL glow curves deconvoluted using the CGCD method. The sample that was not heat-treated showed six overlapping peaks, as shown in Fig. 7(a). After undergoing the first and second thermal treatments, the resulting zinc sulphide sample was exhibited in Fig. 7(b, c) with seven overlapping peaks. For the third and fourth treatments, the number of peaks climbed to eight overlapping peaks, as seen in Fig. 7(d, e). Table 2 provided the average values of the kinetic parameters, E (eV), T_M_ (K), s (s^− 1^), and b for the sample without the thermal treatment and for all the fourth thermal treatments. A radiative transition occurs when a trapped charge carrier is liberated from the matching trap, and we refer to this energy as the activation energy (E). The frequency factor called the attempt-to-escape factor (s) is linked to the possibility of the trapped charge carriers being thermally freed. The first, second, or general-order behaviour of the trapping center is indicated by the order of kinetics (b).

As a result, it was established that only six locations of the produced zinc sulphide sample without thermal treatment for TL components were 358.5, 376.2, 405.3, 431.7, 513.3, and 547.8 K. After undergoing the first thermal treatment, the peak position from one to six exhibits a shift toward the low temperature and appears to peak at 529.9 K. The second treatment’s impact was a change in the peak position to the side with lower temperatures and still seven positions. In addition to creating eight peak locations in the third thermal treatment, additional thermal treatment resulted in the peak site migrating to a lower temperature, with peak eight emerging at 545.7 K. The fourth thermal treatment results in the eight peak positions appearing as well, with significant effort shifting in the peak locations to be 356.1, 364.4, 389.4, 420.7, 448.6, 478.7, 508. 5, and 528.9 K.

**Table 2:** Kinetic parameters of the prepared zinc sulphide samples from the CGCD method before thermal treatment and for all the fourth thermal treatment

		P1	P2	P3	P4	P5	P6	P7	P8
before thermal treatment	**Tm (K)**	358.5	376.2	405.3	431.7	513.3	547.8		
	**E (eV)**	0.98	1.04	1.17	1.31	1.34	1.46		
	**s (s** ^**− 1**^ ) * 10 ^**14**^	0.283	0.312	0.0792	8.3	4.2	1.23		
	**b**	1.53	1.69	1.72	1.71	1.55	1.44		
1st thermal treatment	**Tm (K)**	358.2	375.3	403.3	430.9	460.1	502.9	**529.9**	
	**E (eV)**	1.00	1.05	1.19	1.30	1.36	1.44	**1.55**	
	**s (s** ^**− 1**^ ) * 10 ^**14**^	2.6	0.559	2.29	2.25	0.465	10.01	**1.87**	
	**b**	1.42	1.62	1.82	1.59	1.36	1.51	**1.40**	
2nd thermal treatment	**Tm (K)**	357.5	373.7	402.4	424.7	455.6	489.8	**516.2**	
	**E (eV)**	1.02	1.04	1.17	1.29	1.31	1.47	**1.54**	
	**s (s** ^**− 1**^ ) * 10 ^**14**^	0.855	0.683	1.67	2.59	2.42	20.42	**3.19**	
	**b**	1.51	1.63	1.84	1.57	1.37	1.52	**1.42**	
3rd thermal treatment	**Tm (K)**	357.2	370.94	390.1	422.7	451.0	480.5	**489.7**	**545.7**
	**E (eV)**	0.99	1.01	1.16	1.29	1.31	1.41	**1.49**	**1.57**
	**s (s** ^**− 1**^ ) * 10 ^**14**^	0.446	3.28	4.04	4.07	5.94	10.03	**7.82**	**0.237**
	**b**	1.52	1.59	1.84	1.59	1.31	1.51	**1.40**	**1.41**
4th thermal treatment	**Tm (K)**	356.1	364.4	389.4	420.7	448.6	478.7	**508.5**	**528.9**
	**E (eV)**	0.95	1.01	1.14	1.31	1.36	1.42	**1.51**	**1.56**
	**s (s** ^**− 1**^ ) * 10 ^**14**^	0.0534	0.191	2.11	20.28	7.41	2.90	**3.11**	**6.37**
	**b**	1.55	1.70	1.79	1.00	1.38	1.65	**1.37**	**1.46**

Using thermally treated zinc sulphide samples, it was observed that there was a slight shift in locations from peak one to peak three and a significant shift in positions from peak four to peak eight. Finally, heat treatment produced a trap by shifting the trap position to the side with a lower temperature. Two techniques are used to determine the activation energies: the CGCD method and the experimental IR method. For all TL glow peaks, the activation energy values obtained using the two techniques agree well as listed in Table 3.

**Table 3:** The average values of the activation energy are obtained by the CGCD and IR methods

Peak #	Activation Energy by CGCD Method	Activation Energy by IR Method	Average Activation Energy
P1	0.95	0.82	0.89 ± 0.09
P2	1.01	1.01	1.01 ± 0.00
P3	1.14	1.09	1.12 ± 0.03
P4	1.31	1.28	1.29 ± 0.02
P5	1.36	1.36	1.36 ± 0.00
P6	1.42	1.42	1.42 ± 0.00
P7	1.51	1.51	1.51 ± 0.00
P8	1.56	1.61	1.59 ± 0.04

## Conclusions

The present experiment aims to explore the influence of heat treatment on the luminescent properties and phase transformation of zinc sulphide. The microscopy and X-ray diffraction analysis confirmed the presence of hexagonal structure in the annealed samples in addition to the cubic zinc blend phase. The hexagonal phase percentage was found to change with the increase of heat exposure time as it was found to be at its lowest for the 8 h annealed samples. The presence of both phases enhanced the luminescence of the material; however, the best composition for luminescence among the prepared samples was the 8.2% hexagonal phase and 91.8% cubic phase. The TEM images of the heat-treated samples displayed an aggregated ZnS nanoparticles with almost uniform shape and size. It can be observed that the particles are close to the spherical shape, which in turn confirms the new hexagonal phase formation as determined from x ray diffraction data. The TL-intensities indicated extremely higher thermoluminescence properties of thermally treated ZnS than that of the untreated ones. The analysis of the TL signals revealed the composition of the glow curves of the treated for 8 h at 500^o^C of eight TL components revealed higher TL sensitivity than other treatments. So, ZnS samples that treated thermally for 8 h at 500 ^o^C recommended for studying the TL dosimetric properties. In the future, distortion of the hexagonal additional phase could be studied, which in turn will affect the trap concentration found in the band gap and hence study their influence on the structural and spectroscopic properties.

## Data Availability

No datasets were generated or analysed during the current study.
